# Carroll County Medical Society

**Published:** 1869-02

**Authors:** J. Haller

**Affiliations:** Sec’y.


Society adjourned.

### CARROLL COUNTY MEDICAL SOCIETY.

Pursuant to a call, circulated among the physicians belonging to the regular profession of medicine and surgery, in Carroll County, a meeting was held in the First Baptist Church, in Lanark, on Tuesday, December 8th, 1868, for the purpose of organizing a County Medical Society, for a more thorough protection, both in and out of the profession, against the evils of medical empiricism; and to afford mutual assistance in the amelioration of the sufferings of humanity.

Dr. J. L. Hostetter was chosen temporary Chairman of the meeting. The Constitution, By-Laws, and Rules of Order were then read, and, on motion, adopted as the basis for the government of the Society. The officers for the coming year were then elected, as follows:—

President, Dr. John L. Hostetter, of Mount Carroll; Vice-President, Dr. J. B. Porter, of Lanark; Secretary and Treasurer, Dr. J. Haller, of Lanark.

The following-named members were, by resolution, requested to read papers at the next meeting of the Society, to wit:—On the Action of Chloroform as an Anæsthetic Agent, Dr. N. Stephenson, of Thomson. On the Action of Veratrum Viride sa an Arterial and Nervous Sedative, and to what Extent in Dose it may be safely administered, Dr. D. M. Greeley, of Mount Carroll. On Fractures in General, both Compound and Simple, Dr. B. P. Miller, of Mount Carroll. On the Action of

Gelseminum Sempervirens, Dr. J. Haller, of Lanark. On the History, Rise, and Progress of Medicine, Dr. J. B. Porter, of Lanark. On Medical Ethics in General, Dr. J. L. Hostetter, of Mount Carroll.

On motion, it was resolved that any member of the press and clergymen, who are favorable to the "Regular System of Medicine," be allowed seats at the meetings of this Society.

On motion, it was ordered that the Secretary make out and forward a report of this meeting to the CHICAGO MEDICAL EXAMINER and *Chicago Medical Journal*, for publication.

There was a good attendance of the physicians of the county, and a most cordial feeling prevailed. After an interchange of views on different topics of medicine, the Society adjourned, to meet in Lanark, on Monday, Feb. 22, 1869, at 2 o'clock P.M.

J. HALLER, M.D., *Sec'y.*

